# Implicit Contractive Mappings in Modular Metric and Fuzzy Metric Spaces

**DOI:** 10.1155/2014/981578

**Published:** 2014-06-05

**Authors:** N. Hussain, P. Salimi

**Affiliations:** ^1^Department of Mathematics, King Abdulaziz University, P.O. Box 80203, Jeddah 21589, Saudi Arabia; ^2^Young Researchers and Elite Club, Rasht Branch, Islamic Azad University, Rasht, Iran

## Abstract

The notion of modular metric spaces being a natural generalization of classical modulars over linear spaces like Lebesgue, Orlicz, Musielak-Orlicz, Lorentz, Orlicz-Lorentz, and Calderon-Lozanovskii spaces was recently introduced. In this paper we investigate the existence of fixed points of generalized *α*-admissible modular contractive mappings in modular metric spaces. As applications, we derive some new fixed point theorems in partially ordered modular metric spaces, Suzuki type fixed point theorems in modular metric spaces and new fixed point theorems for integral contractions. In last section, we develop an important relation between fuzzy metric and modular metric and deduce certain new fixed point results in triangular fuzzy metric spaces. Moreover, some examples are provided here to illustrate the usability of the obtained results.

## 1. Introduction and Basic Definitions

Chistyakov introduced the notion of modular metric spaces in [1, 2]. The main idea behind this new concept is the physical interpretation of the modular. Informally speaking, whereas a metric on a set represents nonnegative finite distances between any two points of the set, a modular on a set attributes a nonnegative (possibly, infinite valued) “field of (generalized) velocities”: to each “time” *λ* > 0 the absolute value of an average velocity *ω*_*λ*_(*x*, *y*) is associated in such a way that in order to cover the “distance” between points *x*, *y* ∈ *X* it takes time *λ* to move from *x* to *y* with velocity *ω*_*λ*_(*x*, *y*). But the way we approached the concept of modular metric spaces is different. Indeed we look at these spaces as the nonlinear version of the classical modular spaces introduced by Nakano [3] on vector spaces and modular function spaces introduced by Musielak [4] and Orlicz [5, 6].

For the study of electrorheological fluids (for instance lithium polymethacrylate), modeling with sufficient accuracy using classical Lebesgue and Sobolev spaces, *L* ^*p*^ and *W* ^1,*p*^, where *p* is a fixed constant is not adequate, but rather the exponent *p* should be able to vary [7]. One of the most interesting problems in this setting is the famous Dirichlet energy problem [8, 9]. The classical technique used so far in studying this problem is to convert the energy functional, naturally defined by a modular, to a convoluted and complicated problem which involves a norm (the Luxemburg norm). The modular metric approach is more natural and has not been used extensively. In recent years, there was a strong interest to study the fixed point property in modular function spaces after the first paper [10] was published in 1990. For more on metric fixed point theory, the reader may consult the book [11] and for modular function spaces [12, 13].

Let *X* be a nonempty set. Throughout this paper for a function *ω* : (0, *∞*) × *X* × *X* → [0, *∞*], we will write

(1) $$\begin{array}{c} \omega_{\lambda }\left(x,y\right)=\omega \left(\lambda ,x,y\right), \end{array}$$

for all *λ* > 0 and *x*, *y* ∈ *X*.

**Definition 1 (see [1, 2])**

A function *ω* : (0, *∞*) × *X* × *X* → [0, *∞*] is said to be modular metric on *X* if it satisfies the following axioms:

- *x* = *y* if and only if *ω*_*λ*_(*x*, *y*) = 0, for all *λ* > 0;
- *ω*_*λ*_(*x*, *y*) = *ω*_*λ*_(*y*, *x*), for all *λ* > 0, and *x*, *y* ∈ *X*;
- *ω*_*λ*+*μ*_(*x*, *y*) ≤ *ω*_*λ*_(*x*, *z*) + *ω*_*μ*_(*z*, *y*), for all *λ*, *μ* > 0 and *x*, *y*, *z* ∈ *X*.

If instead of (i), we have only the condition (i′)

(2) $$\begin{array}{c} \omega_{\lambda }\left(x,x\right)=0,\;\forall \lambda >0,\;\;x\in X, \end{array}$$

then *ω* is said to be a pseudomodular (metric) on *X*. A modular metric *ω* on *X* is said to be regular if the following weaker version of (i) is satisfied:

(3) $$\begin{array}{c} x=y\;\text{iff}\;\omega_{\lambda }\left(x,y\right)=0,\;\;\text{for}\;\text{some}\;\lambda >0. \end{array}$$

Finally, *ω* is said to be convex if for *λ*, *μ* > 0 and *x*, *y*, *z* ∈ *X*, it satisfies the inequality

(4) $$\begin{array}{c} \omega_{\lambda +\mu }\left(x,y\right)\leq \frac{\lambda }{\lambda +\mu }\omega_{\lambda }\left(x,z\right)+\frac{\mu }{\lambda +\mu }\omega_{\mu }\left(z,y\right). \end{array}$$

Note that for a metric pseudomodular *ω* on a set *X* and any *x*, *y* ∈ *X*, the function *λ* → *ω*_*λ*_(*x*, *y*) is nonincreasing on (0, *∞*). Indeed, if 0 < *μ* < *λ*, then

(5) $$\begin{array}{c} \omega_{\lambda }\left(x,y\right)\leq \omega_{\lambda -\mu }\left(x,x\right)+\omega_{\mu }\left(x,y\right)=\omega_{\mu }\left(x,y\right). \end{array}$$

Following example presented by Abdou and Khamsi [14] is an important motivation of the concept of modular metric spaces.

**Example 2**

Let *X* be a nonempty set and Σ a nontrivial *σ*-algebra of subsets of *X*. Let *P* be a *δ*-ring of subsets of *X*, such that *E*∩*A* ∈ *P* for any *E* ∈ *P* and *A* ∈ Σ. Let us assume that there exists an increasing sequence of sets *K*_*n*_ ∈ *P* such that *X* = ⋃*K*_*n*_. By *E* we denote the linear space of all simple functions with supports from *P*. By *M*_*∞*_ we will denote the space of all extended measurable functions; that is, all functions *f* : *X* → [−*∞*, *∞*] such that there exists a sequence {*g*_*n*_} ⊂ *E*, |*g*_*n*_| ≤ |*f*|, and *g*_*n*_(*x*) → *f*(*x*) for all *x* ∈ *X*. By 1_*A*_ we denote the characteristic function of the set *A*. Let *ρ* : *M*_*∞*_ → [0, *∞*] be a nontrivial, convex, and even function. We say that *ρ* is a regular convex function pseudomodular if

- *ρ*(0) = 0;
- *ρ* is monotone; that is, |*f*(*x*)| ≤ |*g*(*x*)| for all *x* ∈ *X* implies *ρ*(*f*) ≤ *ρ*(*g*), where *f*, *g* ∈ *M*_*∞*_;
- *ρ* is orthogonally subadditive; that is, *ρ*(*f*1_*A*∪*B*_) ≤ *ρ*(*f*1_*A*_) + *ρ*(*f*1_*B*_) for any *A*, *B* ∈ Σ such that *A*∩*B* ≠ *∅*, *f* ∈ *M*;
- *ρ* has the Fatou property; that is, |*f*_*n*_(*x*)|↑|*f*(*x*)| for all *x* ∈ *X* implies *ρ*(*f*_*n*_)↑*ρ*(*f*), where *f* ∈ *M*_*∞*_;
- *ρ* is order continuous in *E*; that is, *g*_*n*_ ∈ *E* and |*g*_*n*_(*x*)| ↓ 0 implies *ρ*(*g*_*n*_) ↓ 0.

Similarly, as in the case of measure spaces, we say that a set *A* ∈ Σ is *ρ*-null if *ρ*(*g*1_*A*_) = 0 for every *g* ∈ *E*. We say that a property holds *ρ*-almost everywhere if the exceptional set is *ρ*-null. As usual we identify any pair of measurable sets whose symmetric difference is *ρ*-null as well as any pair of measurable functions differing only on a *ρ*-null set. With this in mind we define

(6) $$\begin{array}{c} M\left(X,\Sigma ,P,\rho \right)=\left\{f\in M_{\infty };\left|f\left(x\right)\right|<\infty \rho \text{-a}.\text{e}\right\}, \end{array}$$

where each *f* ∈ *M*(*X*, Σ, *P*, *ρ*) is actually an equivalence class of functions equal to *ρ*-a.e. rather than an individual function. Where no confusion exists we will write *M* instead of *M*(*X*, Σ, *P*, *ρ*). Let *ρ* be a regular function pseudomodular.

- We say that *ρ* is a regular function semimodular if *ρ*(*αf*) = 0 for every *α* > 0 implies *f* = 0  *ρ*-a.e.
- We say that *ρ* is a regular function modular if *ρ*(*f*) = 0 implies *f* = 0  *ρ*-a.e.

The class of all nonzero regular convex function modulars defined on *X* will be denoted by *R*. Let us denote *ρ*(*f*, *E*) = *ρ*(*f*1_*E*_) for *f* ∈ *M*, *E* ∈ Σ. It is easy to prove that *ρ*(*f*, *E*) is a function pseudomodular in the sense of Definition 2.1.1 in [13] (see also [15, 16]). Let *ρ* be a convex function modular.

(a) The associated modular function space is the vector space *L*_*ρ*_(*X*, Σ), or briefly *L*_*ρ*_, defined by
  (7) $$\begin{array}{c} L_{\rho }=\left\{f\in M;\rho \left(\lambda f\right)⟶0\;\text{as}\;\lambda ⟶0\right\}. \end{array}$$
(b) The following formula defines a norm in *L*_*ρ*_ (frequently called Luxemburg norm):
  (8) $$\begin{array}{c} {||f||}_{\rho }=\inf\left\{\alpha >0;\rho \left(\frac{f}{\alpha }\right)\leq 1\right\}. \end{array}$$

A modular function space furnishes a wonderful example of a modular metric space. Indeed, let *L*_*ρ*_ be a modular function space. Define the function modular *ω* by

(9) $$\begin{array}{c} \omega_{\lambda }\left(f,g\right)=\rho \left(\frac{f-g}{\lambda }\right) \end{array}$$

for all *λ* > 0, and *f*, *g* ∈ *L*_*ρ*_. Then *ω* is a modular metric on *L*_*ρ*_. Note that *ω* is convex if and only if *ρ* is convex. Moreover we have

(10) $$\begin{array}{c} {||f-g||}_{\rho }=d_{\omega }^{*}\left(f,g\right), \end{array}$$

for any *f*, *g* ∈ *L*_*ρ*_.

Other easy examples may be found in [1, 2].

**Definition 3**

Let *X*_*ω*_ be a modular metric space.

(1) The sequence (*x*_*n*_)_*n*∈*N*_ in *X*_*ω*_ is said to be *ω*-convergent to *x* ∈ *X*_*ω*_ if and only if for each *λ* > 0, *ω*_*λ*_(*x*_*n*_, *x*) → 0, as *n* → *∞*. *x* will be called the *ω*-limit of (*x*_*n*_).
(2) The sequence (*x*_*n*_)_*n*∈*N*_ in *X*_*ω*_ is said to be *ω*-Cauchy if for each *λ* > 0, *ω*_*λ*_(*x*_*m*_, *x*_*n*_) → 0, as *m*, *n* → *∞*.
(3) A subset *M* of *X*_*ω*_ is said to be *ω*-closed if the *ω*-limit of a *ω*-convergent sequence of *M* always belongs to *M*.
(4) A subset *M* of *X*_*ω*_ is said to be *ω*-complete if any *ω*-Cauchy sequence in *M* is a *ω*-convergent sequence and its *ω*-limit is in *M*.
(5) A subset *M* of *X*_*ω*_ is said to be *ω*-bounded if we have
  (11) $$\begin{array}{c} \delta_{\omega }\left(M\right)=\sup\left\{\omega_{\lambda }\left(x,y\right);x,y\in M\right\}<\infty . \end{array}$$

In 2012, Samet et al. [17] introduced the concepts of *α*-*ψ*-contractive and *α*-admissible mappings and established various fixed point theorems for such mappings defined on complete metric spaces. Afterwards Salimi et al. [18] and Hussain et al. [19–21] modified the notions of *α*-*ψ*-contractive and *α*-admissible mappings and established certain fixed point theorems.

**Definition 4 (see [17])**

Let *T* be self-mapping on *X* and *α* : *X* × *X* → [0, +*∞*) a function. One says that *T* is an *α*-admissible mapping if

(12) $$\begin{array}{c} x,y\in X,\;\alpha \left(x,y\right)\geq 1⟹\alpha \left(Tx,Ty\right)\geq 1. \end{array}$$

**Definition 5 (see [18])**

Let *T* be self-mapping on *X* and *α*, *η* : *X* × *X* → [0, +*∞*) two functions. One says that *T* is an *α*-admissible mapping with respect to *η* if

(13) x,y∈X, α(x,y)≥η(x,y)⟹α(Tx,Ty)               ≥η(Tx,Ty).

Note that if we take *η*(*x*, *y*) = 1 then this definition reduces to Definition 4. Also, if we take, *α*(*x*, *y*) = 1 then we say that *T* is an *η*-subadmissible mapping.

**Definition 6 (see [20])**

Let (*X*, *d*) be a metric space. Let *α*, *η* : *X* × *X* → [0, *∞*) and *T* : *X* → *X* be functions. One says *T* is an *α*-*η*-continuous mapping on (*X*, *d*), if, for given *x* ∈ *X* and sequence {*x*_*n*_} with

(14) xn⟶x  as  n⟶∞, α(xn,xn+1)≥η(xn,xn+1)∀n∈N⟹Txn⟶Tx.

**Example 7 (see [20])**

Let *X* = [0, *∞*) and *d*(*x*, *y*) = |*x* − *y*| be a metric on *X*. Assume *T* : *X* → *X* and *α*, *η* : *X* × *X* → [0, +*∞*) are defined by

(15) $$\begin{array}{c} Tx=\{\begin{array}{cc} x^{5}, & \text{if}\;x\in \left[0,1\right],\\ \sin\pi x+2, & \text{if}\left(1,\infty \right), \end{array}\\ \alpha \left(x,y\right)=\{\begin{array}{cc} x^{2}+y^{2}+1, & \text{if}\;x,y\in \left[0,1\right],\\ 0, & \text{otherwise}\; \end{array} \end{array}$$

and *η*(*x*, *y*) = *x* ^2^. Clearly, *T* is not continuous, but *T* is *α*-*η*-continuous on (*X*, *d*).

A mapping *T* : *X* → *X* is called orbitally continuous at *p* ∈ *X* if lim⁡_*n*→*∞*_⁡*T* ^*n*^ *x* = *p* implies that lim⁡_*n*→*∞*_ *TT* ^*n*^ *x* = *Tp*. The mapping *T* is orbitally continuous on *X* if *T* is orbitally continuous for all *p* ∈ *X*.

**Remark 8 (see [20])**

Let *T* : *X* → *X* be self-mapping on an orbitally *T*-complete metric space *X*. Define *α*, *η* : *X* × *X* → [0, +*∞*) by

(16) $$\begin{array}{c} \alpha \left(x,y\right)=\{\begin{array}{cc} 3, & \text{if}\;x,y\in O\left(w\right)\\ 0, & \text{otherwise}, \end{array}\;\;\eta \left(x,y\right)=1, \end{array}$$

where *O*(*w*) is an orbit of a point *w* ∈ *X*. If *T* : *X* → *X* is an orbitally continuous map on (*X*, *d*), then *T* is *α*-*η*-continuous on (*X*, *d*).

In this paper, we investigate existence and uniqueness of fixed points of generalized *α*-admissible modular contractive mappings in modular metric spaces. As applications, we derive some new fixed point theorems in partially ordered modular metric spaces, Suzuki type fixed point theorems in modular metric spaces and new fixed point theorems for integral contractions. At the end, we develop an important relation between fuzzy metric and modular metric and deduce certain new fixed point results in triangular fuzzy metric spaces. Moreover, some examples are provided here to illustrate the usability of the obtained results.

## 2. Fixed Point Results for Implicit Contractions

Let us first start this section with a definition of a family of functions.

**Definition 9**

Assume that Δ_*H*_ denotes the collection of all continuous functions *H* : *R* ^+^6^^ → *R* satisfying the following.

(*H*1) *H* is increasing in its 1th variable and nonincreasing in its 5th variable.
(*H*2) if *u*, *v* ∈ *R* ^+^ with *u*, *v* > 0 and *H*(*u*, *v*, *v*, *u*, *v* + *u*, 0) ≤ 0, then, there exists *ψ* ∈ Ψ such that
  (17) $$\begin{array}{c} u\leq \psi \left(v\right). \end{array}$$

Notice that here we denote with Ψ the family of nondecreasing functions *ψ* : [0, +*∞*)→[0, +*∞*) such that ∑_*n*=1_^*∞*^ *ψ* ^*n*^(*t*)<+*∞* for all *t* > 0, where *ψ* ^*n*^ is the *n*th iterate of *ψ*.

**Example 10**

Let *H*(*t*_1_, *t*_2_, *t*_3_, *t*_4_, *t*_5_, *t*_6_) = *t*_1_ − *ψ*(max⁡{*t*_2_, (*t*_3_ + *t*_4_)/2, (*t*_5_ + *t*_6_)/2}), where *ψ* ∈ Ψ; then *H* ∈ Δ_*H*_.

**Example 11**

Let *H*(*t*_1_, *t*_2_, *t*_3_, *t*_4_, *t*_5_, *t*_6_) = *t*_1_ − *ψ*(max⁡{*t*_2_, (1 + *t*_3_)*t*_4_/(1 + *t*_2_)}), where *ψ* ∈ Ψ; then *H* ∈ Δ_*H*_.

**Theorem 12**

Let *X*_*ω*_ be a complete modular metric space and *T* : *X*_*ω*_ → *X*_*ω*_ self-mapping satisfying the following assertions:

(i) *T* is an *α*-admissible mapping with respect to *η*;
(ii) there exists *x*_0_ ∈ *X* such that *α*(*x*_0_, *Tx*_0_) ≥ *η*(*x*_0_, *Tx*_0_);
(iii) *T* is an *α*-*η*-continuous function;
(iv) assume that there exists *H* ∈ Δ_*H*_ such that for all *x*, *y* ∈ *X*_*ω*_ and *λ* > 0 with *η*(*x*, *Tx*) ≤ *α*(*x*, *y*) we have
  (18) H(ωλ/c(Tx,Ty),ωλ/l(x,y),ωλ/l(x,Tx),ωλ/l(y,Ty),   ω2λ/l(x,Ty),ωλ/l(y,Tx))≤0,
 where 0 < *l* < *c*.

Then *T* has a fixed point. Moreover, if for all *x*, *y* ∈ Fix⁡⁡(*T*) we have *η*(*x*, *x*) ≤ *α*(*x*, *y*) and *H*(*u*, *u*, 0,0, *u*, *u*) > 0 for all *u* > 0, then *T* has a unique fixed point.

**Proof**

Let *x*_0_ ∈ *X* such that *α*(*x*_0_, *Tx*_0_) ≥ *η*(*x*_0_, *Tx*_0_). For *x*_0_ ∈ *X*, we define the sequence {*x*_*n*_} by *x*_*n*_ = *T* ^*n*^ *x*_0_ = *Tx*_*n*_. Now since *T* is an *α*-admissible mapping with respect to *η* then *α*(*x*_0_, *x*_1_) = *α*(*x*_0_, *Tx*_0_) ≥ *η*(*x*_0_, *Tx*_0_) = *η*(*x*_0_, *x*_1_). By continuing this process we have

(19) $$\begin{array}{c} \eta \left(x_{n-1},Tx_{n-1}\right)=\eta \left(x_{n-1},x_{n}\right)\leq \alpha \left(x_{n-1},x_{n}\right) \end{array}$$

for all *n* ∈ *N*. Also, let there exists *n*_0_ ∈ *X* such that *x*_*n*_0__ = *x*_*n*_0_+1_. Then *x*_*n*_0__ is fixed point of *T* and we have nothing to prove. Hence, we assume *x*_*n*_ ≠ *x*_*n*+1_ for all *n* ∈ *N* ∪ {0}. Now by taking *x* = *x*_*n*−1_ and *y* = *x*_*n*_ in (iv) we get

(20) H(ωλ/c(Txn−1,Txn),ωλ/l(xn−1,xn),ωλ/l(xn−1,Txn−1),   ωλ/l(xn,Txn),ω2λ/l(xn−1,Txn),ωλ/l(xn,Txn−1))≤0

which implies

(21) H(ωλ/c(xn,xn+1),ωλ/l(xn−1,xn),ωλ/l(xn−1,xn),   ωλ/l(xn,xn+1),ω2λ/l(xn−1,xn+1),0)≤0.

On the other hand,

(22) ω2λ/l(xn−1,xn+1)≤ωλ/l(xn−1,xn)+ωλ/l(xn,xn+1)≤ωλ/l(xn−1,xn)+ωλ/c(xn,xn+1),ωλ/l(xn,xn+1)≤ωλ/c(xn,xn+1).

Now since *H* is nonincreasing in its 5th variable, so by (21) and (22) we obtain

(23) H(ωλ/c(xn,xn+1),ωλ/l(xn−1,xn),ωλ/l(xn−1,xn),   ωλ/c(xn,xn+1),ωλ/l(xn−1,xn)   +ωλ/c(xn,xn+1),0)≤0.

From (*H*1) we deduce that

(24) $$\begin{array}{c} \omega_{\lambda /c}\left(x_{n},x_{n+1}\right)\leq \psi \left(\omega_{\lambda /l}\left(x_{n-1},x_{n}\right)\right)\leq \psi \left(\omega_{\lambda /c}\left(x_{n-1},x_{n}\right)\right). \end{array}$$

Inductively, we obtain

(25) $$\begin{array}{c} \omega_{\lambda /c}\left(x_{n},x_{n+1}\right)\leq \psi^{n}\left(\omega_{\lambda /c}\left(x_{0},x_{1}\right)\right). \end{array}$$

Taking limit as *n* → *∞* in the above inequality we get

(26) $$\begin{array}{c} \underset{n\to \infty }{\lim}\omega_{\lambda /c}\left(x_{n},x_{n+1}\right)=0. \end{array}$$

Suppose *m*, *n* ∈ *N* with *m* > *n* and *ϵ* > 0 be given. Then there exists *n*_*λ*/(*m*−*n*)_ ∈ *N* such that

(27) $$\begin{array}{c} \omega_{\lambda /c(m-n)}\left(x_{n},x_{n+1}\right)<\frac{\epsilon }{c\left(m-n\right)} \end{array}$$

for all *n* ≥ *n*_*λ*/(*m*−*n*)_. Therefore we get

(28) ωλ/c(xn,xm)≤ωλ/c(m−n)(xn,xn+1) +ωλ/c(m−n)(xn+1,xn+2)+⋯ +ωλ/c(m−n)(xm−1,xm)<ϵc(m−n)+ϵc(m−n)+ϵc(m−n)+⋯ +ϵc(m−n)=ϵc

for all *m*, *n* ≥ *n*_*λ*/(*m*−*n*)_. This shows that {*x*_*n*_} is a Cauchy sequence. Since *X*_*ω*_ is complete, so there exists x* ∈ *X*_*ω*_ such that lim⁡_*n*→*∞*_ *x*_*n*_ = x*. Now since *T* is an *α*-*η*-continuous mapping, so *Tx*_*n*_ → Tx* as *n* → *∞*. Therefore,

(29) $$\begin{array}{c} Tx^{*}=\underset{n\to \infty }{\lim}Tx_{n}=\underset{n\to \infty }{\lim}x_{n+1}=x^{*}. \end{array}$$

Thus *T* has a fixed point. Let all *x*, *y* ∈ Fix⁡(*T*) we have *η*(*x*, *x*) ≤ *α*(*x*, *y*) and *H*(*u*, *u*, 0,0, *u*, *u*) > 0 for all *u* > 0. Then by (iv)

(30) H(ωλ/l(x,y),ωλ/l(x,y),0,0,ωλ/l(x,y),ωλ/l(y,x))  ≤H(ωλ/c(x,y),ωλ/l(x,y),0,0,      ω2λ/l(x,y),ωλ/l(y,x))  =H(ωλ/c(Tx,Ty),ωλ/l(x,y),ωλ/l(x,Tx),      ωλ/l(y,Ty),ω2λ/l(x,Ty),ωλ/l(y,Tx))≤0.

Now if *ω*_*λ*/*l*_(*x*, *y*) > 0, then

(31) 0<H(ωλ/l(x,y),ωλ/l(x,y),0,0,     ωλ/l(x,y),ωλ/l(y,x))≤0

which is a contradiction. Hence, *ω*_*λ*/*l*_(*x*, *y*) = 0. That is, *x* = *y*. Thus *T* has a unique fixed point.

**Corollary 13**

Let *X*_*ω*_ be a complete modular metric space and *T* : *X*_*ω*_ → *X*_*ω*_ a self-mapping satisfying the following assertions:

(i) *T* is an *α*-admissible mapping;
(ii) there exists *x*_0_ ∈ *X* such that *α*(*x*_0_, *Tx*_0_) ≥ 1;
(iii) *T* is an *α*-continuous function;
(iv) assume that there exists *H* ∈ Δ_*H*_ such that for all *x*, *y* ∈ *X*_*ω*_ and *λ* > 0 with *α*(*x*, *y*) ≥ 1 we have
  (32) H(ωλ/c(Tx,Ty),ωλ/l(x,y),ωλ/l(x,Tx),ωλ/l(y,Ty),   ω2λ/l(x,Ty),ωλ/l(y,Tx))≤0,
 where 0 < *l* < *c*.

Then *T* has a fixed point. Moreover, if for all *x*, *y* ∈ Fix⁡⁡(*T*) we have 1 ≤ *α*(*x*, *y*) and *H*(*u*, *u*, 0,0, *u*, *u*) > 0 for all *u* > 0, then *T* has a unique fixed point.

**Theorem 14**

Let *X*_*ω*_ be a complete modular metric space and *T* : *X*_*ω*_ → *X*_*ω*_ self-mapping satisfying the following assertions:

(i) *T* is an *α*-admissible mapping with respect to *η*;
(ii) there exists *x*_0_ ∈ *X* such that *α*(*x*_0_, *Tx*_0_) ≥ *η*(*x*_0_, *Tx*_0_);
(iii) if {*x*_*n*_} is a sequence in *X* such that *α*(*x*_*n*_, *x*_*n*+1_) ≥ *η*(*x*_*n*_, *x*_*n*+1_) with *x*_*n*_ → *x* as *n* → *∞*, then either
  (33) η(Txn,T2xn)≤α(Txn,x)  orη(T2xn,T3xn)≤α(T2xn,x)
 holds for all *n* ∈ *N*;
(iv) condition (iv) of Theorem 12 holds.

Then *T* has a fixed point. Moreover, if for all *x*, *y* ∈ Fix⁡(*T*) we have *η*(*x*, *x*) ≤ *α*(*x*, *y*) and *H*(*u*, *u*, 0,0, *u*, *u*) > 0 for all *u* > 0, then *T* has a unique fixed point.

**Proof**

As in proof of Theorem 12, we can deduce a sequence {*x*_*n*_} such that *x*_*n*+1_ = *Tx*_*n*_ with *α*(*x*_*n*_, *x*_*n*+1_) ≥ *η*(*x*_*n*_, *x*_*n*+1_) and there exists x* ∈ *X*_*ω*_ such that *x*_*n*_ → x* as *n* → *∞*. From (iii) either

(34) η(xn,Txn)≤α(xn,x∗)  orη(xn+1,Txn+1)≤α(xn+1,x∗)

holds for all *n* ∈ *N*. Let *η*(*x*_*n*_, *Tx*_*n*_) ≤ *α*(*x*_*n*_, x*). Then by taking *x* = *x*_*n*_ and *y* = x* in (iv) we have

(35) H(ωλ/c(Txn,Tx∗),ωλ/l(xn,x∗),ωλ/l(xn,Txn),   ωλ/l(x∗,Tx∗),ω2λ/l(xn,Tx∗),ωλ/l(x∗,Txn))≤0

which implies

(36) H(ωλ/c(xn+1,Tx∗),ωλ/l(xn,x∗),   ωλ/l(xn,xn+1),ωλ/l(x∗,Tx∗),   ω2λ/l(xn,Tx∗),ωλ/l(x∗,xn+1))≤0.

Taking limit as *n* → *∞* in the above inequality we obtain

(37) H(ωλ/c(x∗,Tx∗),0,0,   ωλ/l(x∗,Tx∗),ω2λ/l(x∗,Tx∗),0)≤0.

Now since, *ω*_2*λ*/*l*_(x*, Tx*) < *ω*_*λ*/*l*_(x*, Tx*), *ω*_*λ*/*l*_(x*, Tx*) < *ω*_*λ*/*c*_(x*, Tx*), *H* is increasing in its 1th variable and nonincreasing in its 5th variable, so we obtain

(38) H(ωλ/l(x∗,Tx∗),0,0,   ωλ/l(x∗,Tx∗),ωλ/l(x∗,Tx∗),0)≤0

which is a contradiction. Now by taking *u* = *ω*_*λ*/*l*_(x*, Tx*) and *v* = 0, from (*H*2) we have

(39) $$\begin{array}{c} \omega_{\lambda /l}\left(x^{*},Tx^{*}\right)\leq \psi \left(0\right)=0. \end{array}$$

Hence, *ω*_*λ*/*l*_(x*, Tx*) = 0; that is, x* = Tx*. Similarly we can deduce that Tx* = x* when *η*_*λ*_(*x*_*n*+1_, *Tx*_*n*+1_) ≤ *α*_*λ*_(*x*_*n*+1_, x*).

By using Example 10 and Theorem 14 we can obtain the following corollary.

**Corollary 15**

Let *X*_*ω*_ be a complete modular metric space. Let *T* : *X*_*ω*_ → *X*_*ω*_ be self-mapping satisfying the following assertions:

(i) *T* is an *α*-admissible mapping with respect to *η*;
(ii) there exists *x*_0_ ∈ *X* such that *α*(*x*_0_, *Tx*_0_) ≥ *η*(*x*_0_, *Tx*_0_);
(iii) if {*x*_*n*_} is a sequence in *X* such that *α*(*x*_*n*_, *x*_*n*+1_) ≥ *η*(*x*_*n*_, *x*_*n*+1_) with *x*_*n*_ → *x* as *n* → *∞*, then either
  (40) η(Txn,T2xn)≤α(Txn,x)  orη(T2xn,T3xn)≤α(T2xn,x)
 holds for all *n* ∈ *N*;
(iv) for all *x*, *y* ∈ *X*_*ω*_ and *λ* > 0 with *η*(*x*, *Tx*) ≤ *α*(*x*, *y*) we have
  (41) ωλ/c(Tx,Ty)≤ψ(max⁡{ωλ/l(x,y),      ωλ/l(x,Tx)+ωλ/l(y,Ty)2,      ω2λ/l(x,Ty)+ωλ/l(y,Tx)2}),
 where *ψ* ∈ Ψ and 0 < *l* < *c*.

Then *T* has a fixed point. Moreover, if for all *x*, *y* ∈ Fix⁡⁡(*T*) we have *η*(*x*, *x*) ≤ *α*(*x*, *y*), then *T* has a unique fixed point.

**Corollary 16**

Let *X*_*ω*_ be a complete modular metric space. Let *T* : *X*_*ω*_ → *X*_*ω*_ be self-mapping satisfying the following assertions:

(i) *T* is an *α*-admissible mapping with respect to *η*;
(ii) there exists *x*_0_ ∈ *X* such that *α*(*x*_0_, *Tx*_0_) ≥ *η*(*x*_0_, *Tx*_0_);
(iii) if {*x*_*n*_} is a sequence in *X* such that *α*(*x*_*n*_, *x*_*n*+1_) ≥ *η*(*x*_*n*_, *x*_*n*+1_) with *x*_*n*_ → *x* as *n* → *∞*, then either
  (42) η(Txn,T2xn)≤α(Txn,x)  orη(T2xn,T3xn)≤α(T2xn,x)
 holds for all *n* ∈ *N*;
(iv) for all *x*, *y* ∈ *X*_*ω*_ and *λ* > 0 with *η*(*x*, *Tx*) ≤ *α*(*x*, *y*) we have
  (43) ωλ/c(Tx,Ty) ≤ψ(max⁡{ωλ/l(x,y),[1+ωλ/l(x,Tx)]ωλ/l(y,Ty)1+ωλ/l(x,y)}),
 where *ψ* ∈ Ψ and 0 < *l* < *c*.

Then *T* has a fixed point. Moreover, if for all *x*, *y* ∈ Fix⁡⁡(*T*) we have *η*(*x*, *x*) ≤ *α*(*x*, *y*), then *T* has a unique fixed point.

**Corollary 17**

Let *X*_*ω*_ be a complete modular metric space. Let *T* : *X*_*ω*_ → *X*_*ω*_ be self-mapping satisfying the following assertions:

(i) *T* is an *α*-admissible mapping with respect to *η*;
(ii) there exists *x*_0_ ∈ *X* such that *α*(*x*_0_, *Tx*_0_) ≥ *η*(*x*_0_, *Tx*_0_);
(iii) if {*x*_*n*_} is a sequence in *X* such that *α*(*x*_*n*_, *x*_*n*+1_) ≥ *η*(*x*_*n*_, *x*_*n*+1_) with *x*_*n*_ → *x* as *n* → *∞*, then either
  (44) η(Txn,T2xn)≤α(Txn,x)  orη(T2xn,T3xn)≤α(T2xn,x)
 holds for all *n* ∈ *N*;
(iv) for all *x*, *y* ∈ *X*_*ω*_ and *λ* > 0 with *η*(*x*, *Tx*) ≤ *α*(*x*, *y*) we have
  (45) ωλ/c(Tx,Ty)≤aωλ/l(x,y) +b[1+ωλ/l(x,Tx)]ωλ/l(y,Ty)1+ωλ/l(x,y),
 where *a* + *b* < 1 and 0 < *l* < *c*.

Then *T* has a fixed point. Moreover, if for all *x*, *y* ∈ Fix⁡⁡(*T*) we have *η*(*x*, *x*) ≤ *α*(*x*, *y*), then *T* has a unique fixed point.

**Example 18**

Let *X*_*ω*_ = [0, +*∞*) and *ω*_*λ*_(*x*, *y*) = (1/*λ*)|*x* − *y*|. Define *T* : *X*_*ω*_ → *X*_*ω*_,  *α*, *η* : *X*_*ω*_ × *X*_*ω*_ → [0, *∞*), and *ψ* : [0, *∞*)→[0, *∞*) by

(46) $$\begin{array}{c} Tx=\{\begin{array}{cc} \frac{1}{16}x^{2}, & \text{if}\;x\in \left[0,1\right],\\ \frac{\sin^{2}x+\cos x+1}{\sqrt{x^{2}+1}}, & \text{if}\;x\in \left(1,2\right],\\ 7x+\ln x, & \text{if}\;x\in \left(2,\infty \right), \end{array}\\ \alpha \left(x,y\right)=\{\begin{array}{cc} \frac{1}{2}, & \text{if}\;x,y\in \left[0,1\right],\\ \frac{1}{8}, & \text{otherwise}, \end{array}\\ \eta \left(x,y\right)=\frac{1}{4},\;\;\psi \left(t\right)=\frac{1}{2}t. \end{array}$$

Let *α*(*x*, *y*) ≥ *η*(*x*, *y*); then *x*, *y* ∈ [0,1]. On the other hand, *Tw* ∈ [0,1] for all *w* ∈ [0,1]. Then, *α*(*Tx*, *Ty*) ≥ *η*(*Tx*, *Ty*). That is, *T* is an *α*-admissible mapping with respect to *η*. If {*x*_*n*_} is a sequence in *X* such that *α*(*x*_*n*_, *x*_*n*+1_) ≥ *η*(*x*_*n*_, *x*_*n*+1_) with *x*_*n*_ → *x* as *n* → *∞*, then *Tx*_*n*_, *T* ^2^ *x*_*n*_, *T* ^3^ *x*_*n*_ ∈ [0,1] for all *n* ∈ *N*. That is,

(47) $$\begin{array}{c} \eta \left(Tx_{n},T^{2}x_{n}\right)\leq \alpha \left(Tx_{n},x\right),\\ \eta \left(T^{2}x_{n},T^{3}x_{n}\right)\leq \alpha \left(T^{2}x_{n},x\right) \end{array}$$

hold for all *n* ∈ *N*. Clearly, *α*(0, *T*0) ≥ *η*(0, *T*0). Let *α*(*x*, *y*) ≥ *η*(*x*, *Tx*). Now, if *x* ∉ [0,1] or *y* ∉ [0,1], then 1/8 ≥ 1/4, which is a contradiction. So, *x*, *y* ∈ [0,1]. Therefore,

(48) ωλ/2(Tx,Ty)=2λ116|x2−y2|=18λ|x−y||x+y|≤14λ|x−y|=121λ/(1/2)|x−y|=12ωλ/(1/2)(x,y)=ψ(ωλ/(1/2)(x,y))≤ψ(max⁡{ωλ/l(x,y),     [1+ωλ/l(x,Tx)]ωλ/l(y,Ty)1+ωλ/l(x,y)}).

Hence all conditions of Corollary 16 hold and *T* has a unique fixed point.

Let (*X*_*ω*_, ⪯) be a partially ordered modular metric space. Recall that *T* : *X*_*ω*_ → *X*_*ω*_ is nondecreasing if for all *x*, *y* ∈ *X*, *x*⪯*y*⇒*T*(*x*)⪯*T*(*y*). Fixed point theorems for monotone operators in ordered metric spaces are widely investigated and have found various applications in differential and integral equations (see [20, 22–25] and references therein). From results proved above, we derive the following new results in partially ordered modular metric spaces.

**Theorem 19**

Let (*X*_*ω*_, ⪯) be a complete partially ordered modular metric space and *T* : *X*_*ω*_ → *X*_*ω*_ self-mapping satisfying the following assertions:

(i) *T* is nondecreasing;
(ii) there exists *x*_0_ ∈ *X* such that *x*_0_⪯*Tx*_0_;
(iii) *T* is continuous function;
(iv) assume that there exists *H* ∈ Δ_*H*_ such that for all *x*, *y* ∈ *X*_*ω*_ and *λ* > 0 with *x*⪯*y* we have
  (49) H(ωλ/c(Tx,Ty),ωλ/l(x,y),ωλ/l(x,Tx),ωλ/l(y,Ty),   ω2λ/l(x,Ty),ωλ/l(y,Tx))≤0,
 where 0 < l < *c*.

Then *T* has a fixed point. Moreover, if for all *x*, *y* ∈ Fix⁡⁡(*T*) we have *x*⪯*y* and *H*(*u*, *u*, 0,0, *u*, *u*) > 0 for all *u* > 0, then *T* has a unique fixed point.

**Theorem 20**

Let (*X*_*ω*_, ⪯) be a complete partially ordered modular metric space and *T* : *X*_*ω*_ → *X*_*ω*_ self-mapping satisfying the following assertions:

(i) *T* is nondecreasing;
(ii) there exists *x*_0_ ∈ *X* such that *x*_0_⪯*Tx*_0_;
(iii) if {*x*_*n*_} is a sequence in *X* such that *x*_*n*_⪯*x*_*n*+1_ with *x*_*n*_ → *x* as *n* → *∞*, then either
  (50) $$\begin{array}{c} Tx_{n}⪯x\;or\;T^{2}x_{n}⪯x \end{array}$$
 holds for all *n* ∈ *N*;
(iv) assume that there exists *H* ∈ Δ_*H*_ such that for all *x*, *y* ∈ *X*_*ω*_ and *λ* > 0 with *x*⪯*y* we have
  (51) H(ωλ/c(Tx,Ty),ωλ/l(x,y),ωλ/l(x,Tx),ωλ/l(y,Ty),   ω2λ/l(x,Ty),ωλ/l(y,Tx))≤0,
 where 0 < *l* < *c*.

Then *T* has a fixed point. Moreover, if for all *x*, *y* ∈ Fix⁡⁡(*T*) we have *x*⪯*y* and *H*(*u*, *u*, 0,0, *u*, *u*) > 0 for all *u* > 0, then *T* has a unique fixed point.

**Corollary 21**

Let (*X*_*ω*_, ⪯) be a complete partially ordered modular metric space and *T* : *X*_*ω*_ → *X*_*ω*_ self-mapping satisfying the following assertions:

(i) *T* is nondecreasing;
(ii) there exists *x*_0_ ∈ *X* such that *x*_0_⪯*Tx*_0_;
(iii) if {*x*_*n*_} is a sequence in *X* such that *x*_*n*_⪯*x*_*n*+1_ with *x*_*n*_ → *x* as *n* → *∞*, then either
  (52) $$\begin{array}{c} Tx_{n}⪯x\;or\;T^{2}x_{n}⪯x \end{array}$$
 holds for all *n* ∈ *N*;
(iv) assume that for all *x*, *y* ∈ *X*_*ω*_ and *λ* > 0 with *x*⪯*y* we have
  (53) ωλ/c(Tx,Ty)  ≤ψ(max⁡{ωλ/l(x,y),ωλ/l(x,Tx)+ωλ/l(y,Ty)2,        ω2λ/l(x,Ty)+ωλ/l(y,Tx)2}),
 where *ψ* ∈ Ψ and 0 < *l* < *c*.

Then *T* has a fixed point. Moreover, if for all *x*, *y* ∈ Fix⁡⁡(*T*) we have *x*⪯*y*, then *T* has a unique fixed point.

**Corollary 22**

Let (*X*_*ω*_, ⪯) be a complete partially ordered modular metric space and *T* : *X*_*ω*_ → *X*_*ω*_ self-mapping satisfying the following assertions:

(i) *T* is nondecreasing;
(ii) there exists *x*_0_ ∈ *X* such that *x*_0_⪯*Tx*_0_;
(iii) if {*x*_*n*_} is a sequence in *X* such that *x*_*n*_⪯*x*_*n*+1_ with *x*_*n*_ → *x* as *n* → *∞*, then either
  (54) $$\begin{array}{c} Tx_{n}⪯x\;or\;T^{2}x_{n}⪯x \end{array}$$
 holds for all *n* ∈ *N*;
(iv) assume that for all *x*, *y* ∈ *X*_*ω*_ and *λ* > 0 with *x*⪯*y* we have
  (55) ωλ/c(Tx,Ty) ≤ψ(max⁡{ωλ/l(x,y),[1+ωλ/l(x,Tx)]ωλ/l(y,Ty)1+ωλ/l(x,y)}),
 where *ψ* ∈ Ψ and 0 < *l* < *c*.

Then *T* has a fixed point. Moreover, if for all *x*, *y* ∈ Fix⁡⁡(*T*) we have *x*⪯*y*, then *T* has a unique fixed point.

**Corollary 23**

Let (*X*_*ω*_, ⪯) be a complete partially ordered modular metric space and *T* : *X*_*ω*_ → *X*_*ω*_ self-mapping satisfying the following assertions:

(i) *T* is nondecreasing;
(ii) there exists *x*_0_ ∈ *X* such that *x*_0_⪯*Tx*_0_;
(iii) if {*x*_*n*_} is a sequence in *X* such that *x*_*n*_⪯*x*_*n*+1_ with *x*_*n*_ → *x* as *n* → *∞*, then either
  (56) $$\begin{array}{c} Tx_{n}⪯x\;or\;T^{2}x_{n}⪯x \end{array}$$
 holds for all *n* ∈ *N*;
(iv) assume that for all *x*, *y* ∈ *X*_*ω*_ and *λ* > 0 with *x*⪯*y* we have
  (57) ωλ/c(Tx,Ty)  ≤aωλ/l(x,y)+b[1+ωλ/l(x,Tx)]ωλ/l(y,Ty)1+ωλ/l(x,y),
 where *a* + *b* < 1 and 0 < *l* < *c*.

Then *T* has a fixed point. Moreover, if for all *x*, *y* ∈ Fix⁡⁡(*T*) we have *x*⪯*y*, then *T* has a unique fixed point.

## 3. Suzuki Type Fixed Point Results in Modular Metric Spaces

In 2008, Suzuki proved a remarkable fixed point theorem, that is, a generalization of the Banach contraction principle and characterizes the metric completeness. Consequently, a number of extensions and generalizations of this result appeared in the literature (see [26–30] and references therein). As an application of our results proved above we deduce Suzuki type fixed point theorems in the setting of modular metric spaces.

**Theorem 24**

Let *X*_*ω*_ be a complete modular metric space and *T* continuous self-mapping on *X*. Assume that

(58) ωλ/l(x,Tx)≤ωλ/l(x,y)⟹H(ωλ/c(Tx,Ty),ωλ/l(x,y),    ωλ/l(x,Tx),ωλ/l(y,Ty),    ω2λ/l(x,Ty),ωλ/l(y,Tx))≤0

for all *x*, *y* ∈ *X*_*ω*_ and *λ* > 0, where 0 < *l* < *c*. Then *T* has a fixed point. Moreover, if *H*(*u*, *u*, 0,0, *u*, *u*) > 0 for all *u* > 0, then *T* has a unique fixed point.

**Proof**

Define *α*, *η* : (0, *∞*) × *X*_*ω*_ × *X*_*ω*_ → [0, *∞*) by

(59) $$\begin{array}{c} \alpha \left(x,y\right)=\omega_{\lambda /l}\left(x,y\right),\;\;\eta \left(x,y\right)=\omega_{\lambda /l}\left(x,y\right). \end{array}$$

Clearly, *η*(*x*, *y*) ≤ *α*(*x*, *y*) for all *x*, *y* ∈ *X*_*ω*_ and *λ* > 0. Since *T* is continuous, *T* is *α*-*η*-continuous. Thus conditions (i)–(iii) of Theorem 12 hold. Let *η*(*x*, *Tx*) ≤ *α*(*x*, *y*). Then *ω*_*λ*/*l*_(*x*, *Tx*) ≤ *ω*_*λ*/*l*_(*x*, *y*). So from (58) we obtain

(60) H(ωλ/c(Tx,Ty),ωλ/l(x,y),ωλ/l(x,Tx),ωλ/l(y,Ty),   ω2λ/l(x,Ty),ωλ/l(y,Tx))≤0.

Therefore all conditions of Theorem 12 hold and *T* has a unique fixed point.

**Theorem 25**

Let *X*_*ω*_ be a complete modular metric space and *T* self-mapping on *X*. Assume that

(61) 1−b1−b+aωλ/l(x,Tx)≤ωλ/2l(x,y)⟹ωλ/c(Tx,Ty)≤aωλ/l(x,y) +b[1+ωλ/l(x,Tx)]ωλ/l(y,Ty)1+ωλ/l(x,y)

for all *x*, *y* ∈ *X*_*ω*_ and *λ* > 0, where 0 < *l* < *c* and *a* + *b* < 1. Then *T* has a unique fixed point.

**Proof**

Define *α*, *η* : *X*_*ω*_ × *X*_*ω*_ → [0, *∞*) by

(62) $$\begin{array}{c} \alpha \left(x,y\right)=\omega_{\lambda /2l}\left(x,y\right),\;\;\eta \left(x,y\right)=k\omega_{\lambda /l}\left(x,y\right), \end{array}$$

where *k* = (1 − *b*)/(1 − *b* + *a*). Clearly, *η*(*x*, *y*) ≤ *α*(*x*, *y*) for all *x*, *y* ∈ *X*_*ω*_ and *λ* > 0. Then conditions (i)-(ii) of Corollary 17 hold. Let {*x*_*n*_} be a sequence such that *x*_*n*_ → *x* as *n* → *∞*. Since *kω*_*λ*/*l*_(*Tx*_*n*_, *T* ^2^ *x*_*n*_) ≤ *ω*_*λ*/2*l*_(*Tx*_*n*_, *T* ^2^ *x*_*n*_) for all *n* ∈ *N*, from (61) we obtain

(63) ωλ/l(T2xn,T3xn)  ≤ωλ/c(T2xn,T3xn)  ≤aωλ/l(Txn,T2xn)   +b[1+ωλ/l(Txn,T2xn)]ωλ/l(T2xn,T3xn)1+ωλ/l(Txn,T2xn)  ≤aωλ/l(Txn,T2xn)+bωλ/l(T2xn,T3xn)

which implies

(64) $$\begin{array}{c} \omega_{\lambda /l}\left(T^{2}x_{n},T^{3}x_{n}\right)\leq \frac{a}{1-b}\omega_{\lambda /l}\left(Tx_{n},T^{2}x_{n}\right). \end{array}$$

Suppose there exists *n*_0_ ∈ *N*, such that

(65) η(Txn0,T2xn0)>α(Txn0,x)  orη(T2xn0,T3xn0)>α(T2xn0,x);

then

(66) kωλ/l(Txn0,T2xn0)>ωλ/2l(Txn0,x)  orkωλ/l(T2xn0,T3xn0)>ωλ/2l(T2xn0,x).

Therefore,

(67) ωλ/l(Txn0,T2xn0)  ≤ωλ/2l(Txn0,x)+ωλ/2l(T2xn0,x)  <kωλ/l(Txn0,T2xn0)+kωλ/l(T2xn0,T3xn0)  ≤kωλ/l(Txn0,T2xn0)+ka1−bωλ/l(Txn0,T2xn0)  ≤(k+ka1−b)ωλ/l(Txn0,T2xn0)  =ωλ/l(Txn0,T2xn0)

which is a contradiction. Hence, (iii) of Corollary 17 holds. Thus all conditions of Corollary 17 hold and *T* has a unique fixed point.

**Corollary 26**

Let *X*_*ω*_ be a complete modular metric space and *T* self-mapping on *X*. Assume that

(68) 11+aωλ/l(x,Tx)≤ωλ/2l(x,y)⟹ωλ/c(Tx,Ty)≤aωλ/l(x,y)

for all *x*, *y* ∈ *X*_*ω*_ and *λ* > 0, where 0 < *l* < *c* and *a* < 1. Then *T* has a unique fixed point.

## 4. Fixed Point Results for Integral Type Contractions

Recently, Azadifar et al. [31] and Razani and Moradi [32] proved common fixed point theorems of integral type in modular metric spaces. In this section we present more general fixed point theorems for integral type contractions.

**Theorem 27**

Let *X*_*ω*_ be a complete modular metric space and *T* : *X*_*ω*_ → *X*_*ω*_ self-mapping satisfying the following assertions:

(i) *T* is an *α*-admissible mapping with respect to *η*;
(ii) there exists *x*_0_ ∈ *X* such that *α*(*x*_0_, *Tx*_0_) ≥ *η*(*x*_0_, *Tx*_0_);
(iii) *T* is an *α*-*η*-continuous function;
(iv) assume that there exists *H* ∈ Δ_*H*_ such that for all *x*, *y* ∈ *X*_*ω*_ and *λ* > 0 with *η*(*x*, *Tx*) ≤ *α*(*x*, *y*) we have
  (69) H(∫0ωλ/c(Tx,Ty)ρ(t)dt,∫0ωλ/l(x,y)ρ(t)dt,    ∫0ωλ/l(x,Tx)ρ(t)dt,∫0ωλ/l(y,Ty)ρ(t)dt,    ∫0ω2λ/l(x,Ty)ρ(t)dt,∫0ωλ/l(y,Tx)ρ(t)dt)≤0,
 where 0 < *l* < *c*, *ρ* : [0, *∞*)→[0, *∞*) is a Lebesgue-integrable mapping satisfying ∫_0_^*ɛ*^ *ρ*(*t*)*dt* > 0 for *ɛ* > 0.

Then *T* has a fixed point. Moreover, if for all *x*, *y* ∈ Fix⁡⁡(*T*) we have *η*(*x*, *x*) ≤ *α*(*x*, *y*) and *H*(*u*, *u*, 0,0, *u*, *u*) > 0 for all *u* > 0, then *T* has a unique fixed point.

**Theorem 28**

Let *X*_*ω*_ be a complete modular metric space and *T* : *X*_*ω*_ → *X*_*ω*_ self-mapping satisfying the following assertions:

(i) *T* is an *α*-admissible mapping with respect to *η*;
(ii) there exists *x*_0_ ∈ *X* such that *α*(*x*_0_, *Tx*_0_) ≥ *η*(*x*_0_, *Tx*_0_);
(iii) if {*x*_*n*_} is a sequence in *X* such that *α*(*x*_*n*_, *x*_*n*+1_) ≥ *η*(*x*_*n*_, *x*_*n*+1_) with *x*_*n*_ → *x* as *n* → *∞*, then either
  (70) η(Txn,T2xn)≤α(Txn,x)  orη(T2xn,T3xn)≤α(T2xn,x)
 holds for all *n* ∈ *N*;
(iv) condition (iv) of Theorem 27 holds.

Then *T* has a fixed point. Moreover, if for all *x*, *y* ∈ Fix⁡⁡(*T*) we have *η*(*x*, *x*) ≤ *α*(*x*, *y*) and *H*(*u*, *u*, 0,0, *u*, *u*) > 0 for all *u* > 0, then *T* has a unique fixed point.

**Theorem 29**

Let *X*_*ω*_ be a complete modular metric space and *T* continuous self-mapping on *X*. Assume that

(71) ∫0ωλ/l(x,Tx)ρ(t)dt ≤∫0ωλ/l(x,y)ρ(t)dt ⟹H(∫0ωλ/c(Tx,Ty)ρ(t)dt,∫0ωλ/l(x,y)ρ(t)dt,       ∫0ωλ/l(x,Tx)ρ(t)dt,∫0ωλ/l(y,Ty)ρ(t)dt,       ∫0ω2λ/l(x,Ty)ρ(t)dt,∫0ωλ/l(y,Tx)ρ(t)dt)≤0

for all *x*, *y* ∈ *X*_*ω*_ and *λ* > 0, where 0 < *l* < *c*, *ρ* : [0, *∞*)→[0, *∞*) is a Lebesgue-integrable mapping satisfying ∫_0_^*ɛ*^ *ρ*(*t*)*dt* > 0 for *ɛ* > 0. Then *T* has a fixed point. Moreover, if *H*(*u*, *u*, 0,0, *u*, *u*) > 0 for all *u* > 0, then *T* has a unique fixed point.

**Theorem 30**

Let *X*_*ω*_ be a complete modular metric space and *T* self-mapping on *X*. Assume that

(72) 1−b1−b+a∫0ωλ/l(x,Tx)ρ(t)dt  ≤∫0ωλ/2l(x,y)ρ(t)dt⟹∫0ωλ/c(Tx,Ty)ρ(t)dt  ≤a∫0ωλ/l(x,y)ρ(t)dt   +b[1+∫0ωλ/l(x,Tx)ρ(t)dt]∫0ωλ/l(y,Ty)ρ(t)dt1+∫0ωλ/l(x,y)ρ(t)dt

for all *x*, *y* ∈ *X*_*ω*_ and *λ* > 0, where 0 < *l* < *c*, *a* + *b* < 1 and *ρ* : [0, *∞*)→[0, *∞*) is a Lebesgue-integrable mapping satisfying ∫_0_^*ɛ*^ *ρ*(*t*)*dt* > 0 for *ɛ* > 0. Then *T* has a unique fixed point.

## 5. Modular Metric Spaces to Fuzzy Metric Spaces

In 1988, Grabiec [33] defined contractive mappings on a fuzzy metric space and extended fixed point theorems of Banach and Edelstein in such spaces. Successively, George and Veeramani [34] slightly modified the notion of a fuzzy metric space introduced by Kramosil and Michálek and then defined a Hausdorff and first countable topology on it. Since then, the notion of a complete fuzzy metric space presented by George and Veeramani has emerged as another characterization of completeness, and many fixed point theorems have also been proved (see for more details [35–39] and the references therein). In this section we develop an important relation between modular metric and fuzzy metric and deduce in fixed point results in a triangular fuzzy metric space.

**Definition 31**

A 3-tuple (*X*, *M*, ∗) is said to be a fuzzy metric space if *X* is an arbitrary set, ∗ is a continuous *t*-norm and *M* is fuzzy set on *X* ^2^ × (0, *∞*) satisfying the following conditions, for all *x*, *y*, *z* ∈ *X* and *t*, *s* > 0:

- *M*(*x*, *y*, *t*) > 0;
- *M*(*x*, *y*, *t*) = 1 for all *t* > 0 if and only if *x* = *y*;
- *M*(*x*, *y*, *t*) = *M*(*y*, *x*, *t*);
- *M*(*x*, *y*, *t*)∗*M*(*y*, *z*, *s*) ≤ *M*(*x*, *z*, *t* + *s*);
- *M*(*x*, *y*, .):(0, *∞*)→[0,1] is continuous.

The function *M*(*x*, *y*, *t*) denotes the degree of nearness between *x* and *y* with respect to *t*.

**Definition 32 (see [36])**

Let (*X*, *M*, ∗) be a fuzzy metric space. The fuzzy metric *M* is called triangular whenever

(73) $$\begin{array}{c} \frac{1}{M\left(x,y,t\right)}-1\leq \frac{1}{M\left(x,z,t\right)}-1+\frac{1}{M\left(z,y,t\right)}-1 \end{array}$$

for all *x*, *y*, *z* ∈ *X* and all *t* > 0.

**Lemma 33 (see [33, 35])**

For all *x*, *y* ∈ *X*, *M*(*x*, *y*, ·) is nondecreasing on (0, *∞*).

As an application of Lemma 33, we establish the following important fact that each triangular fuzzy metric on *X* induces a modular metric.

**Lemma 34**

Let (*X*, *M*, ∗) be a triangular fuzzy metric space. Define

(74) $$\begin{array}{c} \omega_{\lambda }\left(x,y\right)=\frac{1}{M\left(x,y,\lambda \right)}-1 \end{array}$$

for all *x*, *y* ∈ *X* and all *λ* > 0. Then *ω*_*λ*_ is a modular metric on *X*.

**Proof**

Let *s*, *t* > 0. Then we get

(75) M(x,y,s)=M(x,y,s)∗1=M(x,y,s)∗M(x,x,t)≤M(x,y,s+t)

for all *x*, *y* ∈ *X* and *s*, *t* > 0. Now, since (*X*, *M*, ∗) is triangular, then we get

(76) ωλ+μ(x,y)=1M(x,y,μ+λ)−1≤1M(x,z,μ+λ)−1+1M(z,y,μ+λ)−1≤1M(x,z,λ)−1+1M(z,y,μ)−1=ωλ(x,z)+ωμ(z,y).

As an application of Lemma 34 and the results proved above we deduce following new fixed point theorems in triangular fuzzy metric spaces.

**Theorem 35**

Let (*X*, *M*, ∗) be a complete triangular fuzzy metric space and *T* : *X* → *X* self-mapping satisfying the following assertions:

(i) *T* is an *α*-admissible mapping with respect to *η*;
(ii) there exists *x*_0_ ∈ *X* such that *α*(*x*_0_, *Tx*_0_) ≥ *η*(*x*_0_, *Tx*_0_);
(iii) *T* is an *α*-*η*-continuous function;
(iv) assume that there exists *H* ∈ Δ_*H*_ such that for all *x*, *y* ∈ *X* and *λ* > 0 with *η*(*x*, *Tx*) ≤ *α*(*x*, *y*) we have
  (77) H(1M(Tx,Ty,λ/c)−1,1M(x,y,λ/l)−1,   1M(x,Tx,λ/l)−1,1M(y,Ty,λ/l)−1,   1M(x,Ty,2λ/l)−1,1M(y,Tx,λ/l)−1)≤0,
 where 0 < *l* < *c*.

Then *T* has a fixed point. Moreover, if for all *x*, *y* ∈ Fix⁡⁡(*T*) we have *η*(*x*, *x*) ≤ *α*(*x*, *y*) and *H*(*u*, *u*, 0,0, *u*, *u*) > 0 for all *u* > 0, then *T* has a unique fixed point.

**Theorem 36**

Let (*X*, *M*, ∗) be a complete triangular fuzzy metric space and *T* : *X* → *X* self-mapping satisfying the following assertions:

(i) *T* is an *α*-admissible mapping with respect to *η*;
(ii) there exists *x*_0_ ∈ *X* such that *α*(*x*_0_, *Tx*_0_) ≥ *η*(*x*_0_, *Tx*_0_);
(iii) if {*x*_*n*_} is a sequence in *X* such that *α*(*x*_*n*_, *x*_*n*+1_) ≥ *η*(*x*_*n*_, *x*_*n*+1_) with *x*_*n*_ → *x* as *n* → *∞*, then either
  (78) η(Txn,T2xn)≤α(Txn,x)  orη(T2xn,T3xn)≤α(T2xn,x)
 holds for all *n* ∈ *N*;
(iv) condition (iv) of Theorem 35 holds.

Then *T* has a fixed point. Moreover, if for all *x*, *y* ∈ Fix⁡⁡(*T*) we have *η*(*x*, *x*) ≤ *α*(*x*, *y*) and *H*(*u*, *u*, 0,0, *u*, *u*) > 0 for all *u* > 0, then *T* has a unique fixed point.

**Theorem 37**

Let (*X*, *M*, ∗, ⪯) be a partially ordered complete triangular fuzzy metric space and *T* : *X* → *X* self-mapping satisfying the following assertions:

(i) *T* is nondecreasing;
(ii) there exists *x*_0_ ∈ *X* such that *x*_0_⪯*Tx*_0_;
(iii) *T* is continuous function;
(iv) assume that there exists *H* ∈ Δ_*H*_ such that for all *x*, *y* ∈ *X* and *λ* > 0 with *x*⪯*y* we have
  (79) H(1M(Tx,Ty,λ/c)−1,1M(x,y,λ/l)−1,   1M(x,Tx,λ/l)−1,1M(y,Ty,λ/l)−1,   1M(x,Ty,2λ/l)−1,1M(y,Tx,λ/l)−1)≤0,
 where 0 < *l* < *c*.

Then *T* has a fixed point. Moreover, if for all *x*, *y* ∈ Fix⁡⁡(*T*) we have *x*⪯*y* and *H*(*u*, *u*, 0,0, *u*, *u*) > 0 for all *u* > 0, then *T* has a unique fixed point.

**Theorem 38**

Let (*X*, *M*, ∗, ⪯) be a partially ordered complete triangular fuzzy metric space and *T* : *X* → *X* self-mapping satisfying the following assertions:

(i) *T* is nondecreasing;
(ii) there exists *x*_0_ ∈ *X* such that *x*_0_⪯*Tx*_0_;
(iii) if {*x*_*n*_} is a sequence in *X* such that *x*_*n*_⪯*x*_*n*+1_ with *x*_*n*_ → *x* as *n* → *∞*, then either
  (80) $$\begin{array}{c} Tx_{n}⪯x\;or\;T^{2}x_{n}⪯x \end{array}$$
 holds for all *n* ∈ *N*;
(iv) assume that there exists *H* ∈ Δ_*H*_ such that for all *x*, *y* ∈ *X*_*ω*_ and 0*λ* > 0 with *x*⪯*y* we have
  (81) H(1M(Tx,Ty,λ/c)−1,1M(x,y,λ/l)−1,   1M(x,Tx,λ/l)−1,1M(y,Ty,λ/l)−1,   1M(x,Ty,2λ/l)−1,1M(y,Tx,λ/l)−1)≤0,
 where 0 < *l* < *c*.

Then *T* has a fixed point. Moreover, if for all *x*, *y* ∈ Fix⁡⁡(*T*) we have *x*⪯*y* and *H*(*u*, *u*, 0,0, *u*, *u*) > 0 for all *u* > 0, then *T* has a unique fixed point.

**Theorem 39**

Let (*X*, *M*, ∗) be a complete triangular fuzzy metric space and *T* continuous self-mapping on *X*. Assume that

(82) 1M(x,Tx,λ/l)−1 ≤1M(x,y,λ/l)−1 ⟹H(1M(Tx,Ty,λ/c)−1,1M(x,y,λ/l)−1,      1M(x,Tx,λ/l)−1,1M(y,Ty,λ/l)−1,      1M(x,Ty,2λ/l)−1,1M(y,Tx,λ/l)−1)≤0

for all *x*, *y* ∈ *X* and *λ* > 0, where 0 < *l* < *c*. Then *T* has a unique fixed point.

**Theorem 40**

Let (*X*, *M*, ∗) be a complete triangular fuzzy metric space and *T* continuous self-mapping on *X*. Assume that

(83) 1−b1−b+a(1M(x,Tx,λ/l)−1)  ≤1M(x,y,λ/2l)−1⟹1M(Tx,Ty,λ/c)−1  ≤a(1M(x,y,λ/l)−1)   +b(1/M(x,Tx,λ/l))[(1/M(y,Ty,λ/l))−1]1/M(x,y,λ/l)

for all *x*, *y* ∈ *X*_*ω*_ and *λ* > 0, where 0 < *l* < *c* and *a* + *b* < 1. Then *T* has a unique fixed point.
